# Innovative spectrofluorimetric determination of vildagliptin based on a “switch off/on” NS-doped carbon dot nanosensor†

**DOI:** 10.1039/d2ra04991k

**Published:** 2022-09-12

**Authors:** Eman A. Elshenawy, Samah F. El-Malla, Sherin F. Hammad, Fotouh R. Mansour

**Affiliations:** Pharmaceutical Analytical Chemistry Department, Faculty of Pharmacy-Tanta University Tanta 31111 Egypt eman.elshenawy@pharm.tanta.edu.eg

## Abstract

A simple, fast, and green one-step microwave pyrolysis approach was proposed for the synthesis of highly fluorescent nitrogen/sulfur-doped carbon dots (NS-CDs). The proposed NS-CDs were prepared in only one minute from citric acid and thiosemicarbazide. In the presence of Cu^2+^, the fluorescence of NS-CDs was significantly quenched (“turn off”) through the formation of a non-fluorescent NS-CDs/Cu^2+^ complex. This designed sensor could be applied for label-free determination of vildagliptin based on the competition between vildagliptin and the functional groups on NS-CDs for Cu^2+^ complexation, and hence NS-CD fluorescence recovery (“turn on”). Under the optimized conditions, the developed probe (NS-CDs/Cu^2+^) demonstrated a good sensing performance for vildagliptin with linearity in the range of 45–240 μM and a detection limit of 13.411 μM. Owing to its sensitivity, this sensor was successfully applied for vildagliptin determination in human urine samples.

## Abstract 2

Microwave synthesis of a switchable sensor as a label-free recognition system for VLD determination. The sensor was applied for VLD determination in dosage form and human urine.

## Graphical abstract

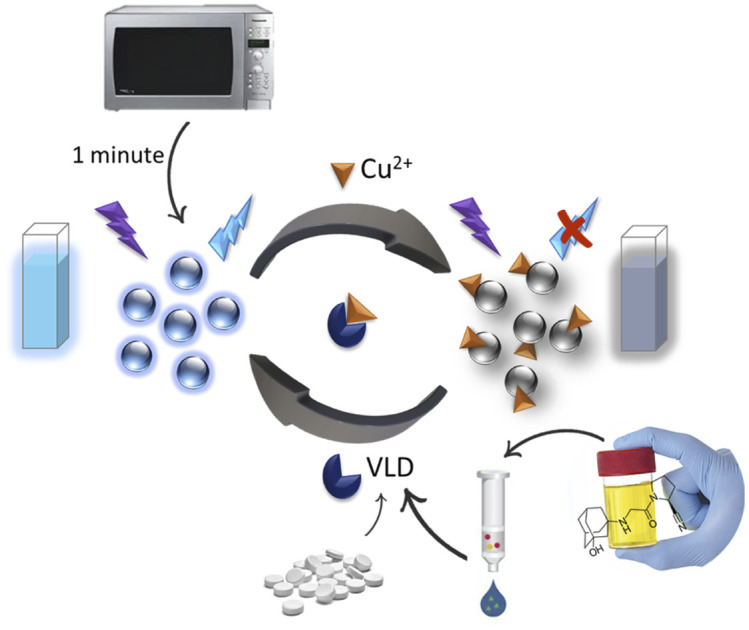

## Introduction

Vildagliptin (VLD) is a potent, selective dipeptidyl peptidase-IV inhibitor that is approved as an oral treatment of type-II diabetes.^1^ VLD acts through the prevention of incretin inactivation, which in turn provokes glucose-dependent insulin release and decreases glucagon levels, thus achieving better glycemic control.^2^ It is effective, with few adverse effects, either alone or in conjunction with other anti-diabetic drugs.^3^ VLD is 20–25% excreted unaltered in urine within 24 hours.^4^

Different methods have been reported for VLD determination including, spectrophotometric,^5–10^ spectrofluorimetric,^11,12^ electrochemical,^13,14^ and chromatographic methods.^15–19^ However, because of the weakly absorbing chromophores in VLD and their significantly blue-shifted maxima, its determination based on direct UV absorption measurement may be prone to interferences from excipients, impurities, or the matrix of biological fluids. The reported spectrofluorimetric methods^11,12^ are based on derivatization reactions that required a long time (20–50 minutes) or temperature. As a result, establishing a VLD detection approach that is quick, inexpensive, and selective is essential.

Carbon dots (CDs), a new member of the carbon nanomaterials family, have lately received great attention in a variety of applications.^20^ CDs demonstrate fascinating properties of excellent water solubility, chemical stability, resistance to photobleaching, the possibility of surface modification, and great biocompatibility which endow them with expanding applications in catalysis, energy storage, biosensing, cellular imaging, and drug targeting.^21–23^ Heteroatom doping of CDs is a reliable and adaptable approach, with N and S being the most widely investigated, for tuning their structural and optical characteristics, and surface reactivity.^24–27^ Among the different techniques used for CD synthesis, microwave irradiation presents various advantages like being easy, environmentally benign, cost-effective, high product yields, and uniform particle size distribution.^27,28^

**Scheme 1:**
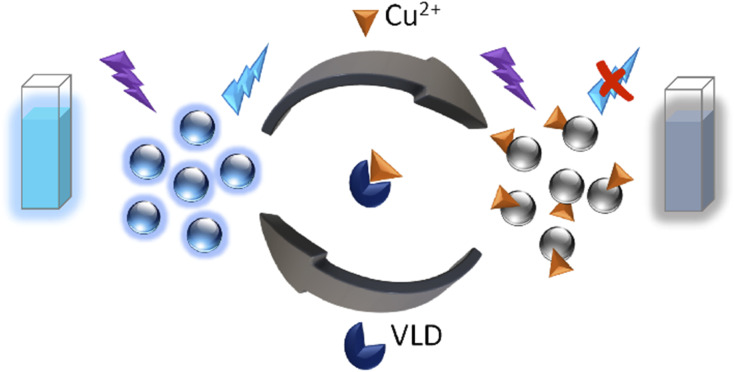
Sensing strategy of the developed probe for VLD determination.

Herein, NS-doped CDs were prepared from citric acid (CA) and thiosemicarbazide (TSC) *via* green one-pot microwave irradiation in only 1 minute. An important feature of this synthesis was being energy-saving, requiring no external heat, and generating CDs with uniform particle size distribution and a high production yield. Furthermore, a carefully designed, rapid, and simple approach based on the as-prepared NS-CDs for the determination of VLD without requiring any chemical reagent was devised. To achieve the aforementioned aim, we developed a switchable sensor by quenching the fluorescence (FL) of NS-CDs with Cu^2+^ through complexation with the functional groups present on NS-CDs surface and then recovering the FL using VLD's ability to complex Cu^2+^(Scheme 1).

## Experimental

### Materials

The starting materials TSC and CA were bought from Alfa Aesar (Germany). Metal salts including CuSO_4_·5H_2_O, NaCl, KCl, MgSO_4_, Co(OAc)_2_, NiSO_4_·6H_2_O, BaCl_2_, Cr_2_(SO_4_)·6H_2_O, Pb(OAc)_2_, ZnCl_2_ were of laboratory grade (ISO-Chem, France). Hydroxy propyl methylcellulose (HPMC), dextrin, lactose monohydrate, glucose, urea, sodium acetate, tartaric acid, boric acid, glacial acetic acid, ammonium acetate, and NaOH were acquired from ADWIC Co. (Egypt). Quinine Sulfate (Alpha Chemika, India) was used as a reference for quantum yield (QY) measurement. Vildagliptin (purity, 99.9%) was offered by Future Pharmaceutical Industries (Egypt). Alogliptin benzoate (99.7%) and saxagliptin (99.0%) were gifted by Global Nabi Pharmaceuticals (6th of October City, Egypt) and Marcyrl Pharmaceuticals Co. (Cairo, Egypt), respectively. Vildagluse® 50 mg tablets were obtained from a community pharmacy. Cleanert® C_18_ cartridge was procured from Agela Technologies (California, USA).

### Apparatus

FL spectra were acquired using a JASCO FP-6300 spectrofluorometer. Shimadzu UV-1800 spectrophotometer was used for obtaining UV absorption spectra. A JEOL JEM-2100 transmission electron microscope with 200 kV accelerating voltage was applied to acquire the morphology and particle size of the NS-CDs as well as the energy-dispersive X-ray analysis (EDX) and selected area electron diffraction (SAED) pattern. Analysis of NS-CDs surface functionality was performed with Jasco 4100 FT/IR spectrophotometer on KBr disc. A 1000 W household microwave oven and Branson 3510 ultrasonic cleaner were used through NS-CDs preparation.

### Preparation of NS-CDs

The NS-CDs were attained by employing a microwave-based synthesis.^29^ Briefly, 0.192 g of CA (1 mmol) and 0.274 g of TSC (3 mmol) were irradiated using a domestic microwave for one minute and a dark orange product was obtained. Then, it was allowed to reach room temp. and was dissolved in distilled water (DW, 20 mL) with ultrasonication for 15 minutes. The attained orange solution was centrifuged (6000 rpm × 15 minutes) prior to filtration with a syringe filter (nylon, 0.22 μm).

### Product yield (PY) and quantum yield (QY) calculation

To calculate PY, the NS-CDs solution was first lyophilized to attain solid NS-CDs that were weighed (*m*_NS-CDs_) and the PY value was estimated employing the equation below :^30^PY = (*m*_NS−CDs_/*m*_s_)× 100where *m*_s_ is the mass of starting materials (TSC and CA).

The QY was measured in comparison to the quinine sulfate (QY_st_ = 0.54) solution in 0.1 M H_2_SO_4_, employing the following equation:QY_*x*_ = QY_st_ (*I*_*x*_/*I*_st_)(*A*_st_/*A*_*x*_)(*η*^2^_*x*_/*η*^2^_st_)where *I*_st_ and *I*_*x*_ are the integrated FL of quinine sulfate and NS-CDs after excitation at 350 nm, respectively, *A*_st_/*A*_*x*_ refers to the absorbance ratio of quinine sulfate to NS-CDs at 350 nm and *η* means the refractive index of the used solvents (*η* = 1.33 for aqueous solvents).^29,31,32^

### Procedure for VLD assay

To 15 mg mL^−1^ NS-CDs (20 μL), various volumes of 0.9 mM VLD stock solution and 200 μL of 1.5 mM Cu^2+^ solution were added, mixed, and completed with DW to 3 mL. FL of the resulting solution was recorded. The selectivity of the proposed method for VLD over possibly interfering materials was tested following the same procedure. All FL recordings were measured at an emission of 430 nm (*λ*_ex_ = 350 nm) while keeping a bandwidth of 5 nm for both excitation and emission monochromators and a scanning speed of 1000 nm min^−1^.

### Tablet and urine sample analysis

For tablet analysis, ten Vildagluse® tablets were powdered and an amount equivalent to 50 mg VLD was transferred to a 100 mL volumetric flask, extracted with 50 mL DW by the aid of sonication (15 minutes) and thereafter completed to the mark with DW. After being filtered through a 0.45 μm filter, 300 μL of the obtained solution was examined by the general steps for VLD determination.

For urine analysis, a urine sample was taken from a healthy volunteer receiving no medication 24 h before sample collection. Urine samples were collected in sterile screw-top containers, the containers were labeled with date and time and stored in the refrigerator without preservatives. The urine sample was maintained at room temperature prior to use. Urine samples were prepared by fortifying 10 mL urine aliquots with 273.1–682.6 μg of VLD. The SPE cartridge was pre-conditioned with 5 mL methanol followed by 5 mL DW. After that, the 10 mL fortified urine aliquot was loaded and washed with 15 mL DW in three portions. VLD was eluted with 1 mL methanol in a 5 mL volumetric flask and completed to mark with DW. Finally, 1 mL of eluted solutions was assayed following the steps for VLD analysis.

## Results and discussion

### Preparation, characterization, and optical performance

The NS-CDs were prepared in a household microwave oven using green single-step pyrolysis in one minute, with a high production yield (64.38 weight percent) and QY of 10.7 percent. The blue luminescent NS-CDs were prepared with easily available starting materials and a simple procedure (Scheme S1†), using CA as a carbon source and TSC as a N/S doping agent.

**Fig. 1:**
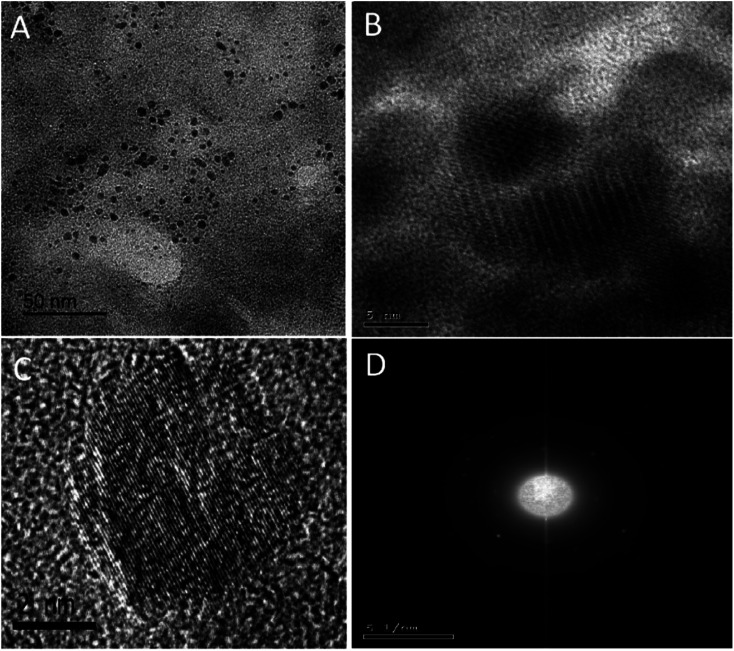
TEM(A), HRTEM (B) of NS-CDs, individual CD showing lattice of 0.20 nm spacing (C) and SAED image of NS-CDs (D).

The produced quasispherical particles NS-CDs were uniformly dispersed with a size range of 1–5 nm, according to TEM images (Fig. 1A). The NS-CDs were found to be crystalline as indicated by lattice fringes (lattice spacing of 0.20 nm) inside the particles^33^ and the pattern observed in SAED image (Fig. 1C and D). To determine the composition of the NS-CDs, EDX was used to conduct the elemental analysis, which revealed that the NS-CDs were primarily composed of carbon, oxygen, nitrogen, and sulfur with 39.94%, 23.03%, 28.56%, and 8.47%, respectively (Fig. S1†), disclosing successful high doping. The functional groups were explored using FT-IR (peaks, cm^−1^); (NH, 3567/3374), (OH, 3203), (CN, 2062), (C=N/N=N, 1497), (C=C, 1620), (C–N/S=O, 1313), (C=O, 1715), (C–O/N–N, 1077), and (C=S, 1417) as can be seen in Fig. S2.†

**Fig. 2:**
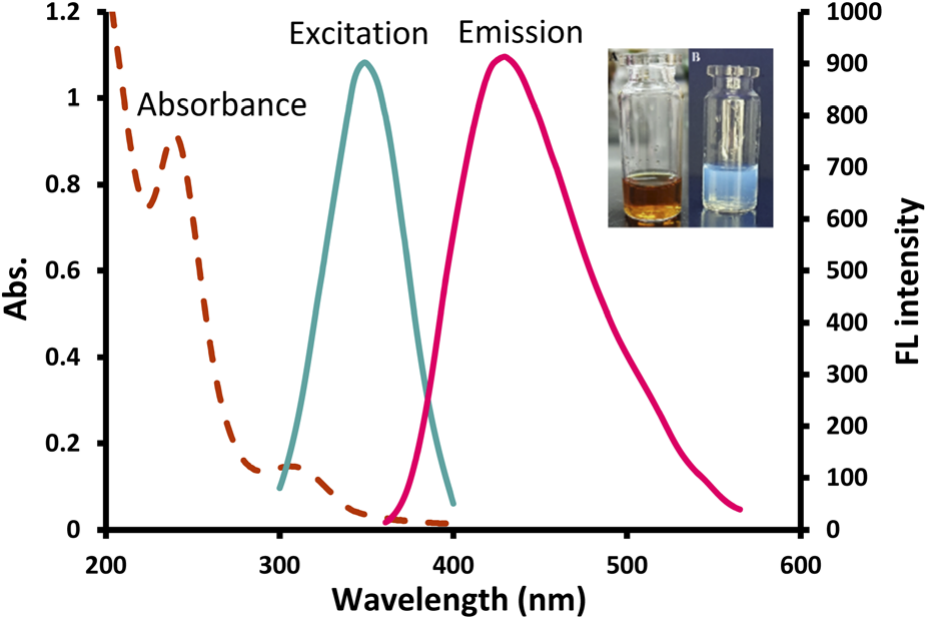
UV absorption, excitation and emission spectra of the prepared NS-CDs; inset: optical image of the prepared NS-CDs under normal light (left) and UV light (right).

The UV spectrum of the prepared CDs (Fig. 2) presented two peaks at 240 nm and 308 nm which correspond to π–π*/n–π* transitions, respectively^34^ and the FL spectrum showed the highest emission at 430 nm following 350 nm excitation (Fig. 2). Quenching was tested in the presence of 100 μM various metal ions and only Cu^2+^ showed a significant quenching effect on the fluorescence intensity (FI) of the NS-CDs probe among the other examined cations. The FL of NS-CDs could be recovered by adding VLD, and consequently, the development of a novel VLD sensor was devised based on the switch off/on FL procedure.

### Optimization of VLD sensing conditions

The sensitivity of the created NS-CD sensor could be affected by experimental conditions such as pH, NS-CDs concentration, the concentration of Cu^2+^, and response time, so they have been investigated and adjusted. Evaluation of the pH effect on the developed sensor was conducted throughout a range of 3.0–10.0 (Fig. S4†). The addition of Cu^2+^ reduced the FI of NS-CDs at all pH values from 3.0 to 10.0 with quenching being higher in acidic media. The highest quenching effect occurred at pH 3.0 with no significant difference from DW. When VLD was added, the highest FI ratio (*F*/*F*_0_ where *F*_0_ is FI of NS-CDs/Cu^2+^ complex while *F* is FI of NS-CDs/Cu^2+^/VLD system) was observed in DW, thus DW was selected for the VLD sensing platform. The effect of NS-CD concentration on FL recovery by VLD was examined over the CDs linearity range (0.01–0.1 mg mL^−1^) and 0.1 mg mL^−1^ NS-CDs resulted in the highest FL recovery. Lower NS-CD concentrations resulted in a lower FL recovery to 0.04 mg mL^−1^ which gave a nearly similar recovery to 0.1 mg mL^−1^ but limited the response range to VLD, so a concentration of 0.1 mg mL^−1^ NS-CDs was chosen to allow better sensitivity (Fig. S5†). In addition, Cu^2+^ concentration impacted the FL recovery produced by VLD as low Cu^2+^ concentrations narrowed the response range to VLD while a very high concentration of Cu^2+^ makes it difficult to recover the FL of NS-CDs, thus Cu^2+^ concentration of 100 μM was selected as the optimum (Fig. S6†). The response of the NS-CDs/Cu^2+^ system to VLD was studied over 20 minutes at minute intervals (Fig. S7†) and the developed sensor showed an immediate response to VLD which persisted for about thirty minutes.

### Assessment of the sensing system process

**Fig. 3:**
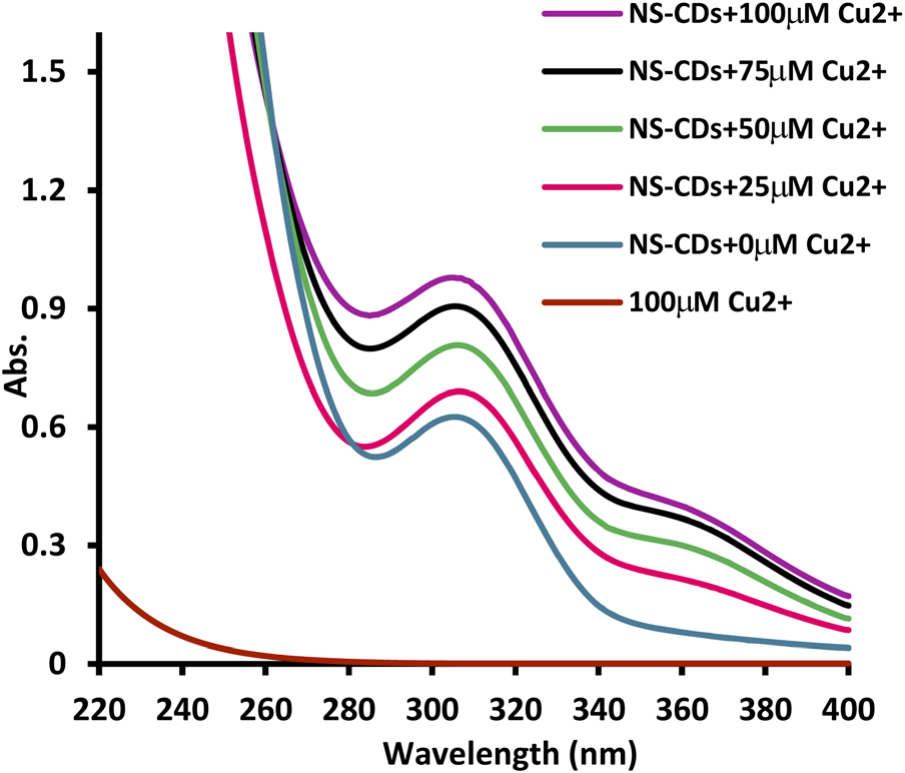
The synthesized NS-CDs UV absorbance spectra with increasing concentration of Cu^2+^.

NS-CDs FL quenching mechanism by Cu^2+^ was studied using the Stern–Volmer equation:*F*_0_/*F* = 1 + *K*_SV_[*Q*]where *F*_0_ and *F* are FIs of NS-CDs and NS-CDs/Cu^2+^ system, while *K*_SV_ and [*Q*] are the Stern–Volmer quenching constant and Cu^2+^ concentration, respectively.^32,35^ At three different temperatures, the FL quenching efficiency (*F*_0_/*F*) was evaluated, and it was found that increasing temperature reduced *K*_SV_ (*K*_SV_ values; 5.88 × 10^9^ L mol^−1^, 6.74 × 10^9^ L mol^−1^, and 7.62 × 10^9^ L mol^−1^, at 313 K, 303 K, and 298 K, respectively) imparting static quenching mechanism^36^ (Fig. S8†). This mechanism includes the formation of NS-CDs/Cu^2+^ non-emissive complex, as evidenced by variations observed in NS-CDs UV spectra after Cu^2+^ addition. When Cu^2+^ was introduced, a new absorption peak was formed (360 nm), which increased with increasing Cu^2+^ concentration, indicating complex formation (Fig. 3). Complexation between NS-CDs and Cu^2+^ occurs through interaction with carboxylic acid, nitrile, or thiocarbonyl groups found on NS-CDs surface.^37,38^ Furthermore, the generated NS-CDs/Cu^2+^ complex absorption peak overlapped with the excitation spectrum of NS-CDs (Fig. S9†), implying the presence of an additional inner filter effect mechanism. This mechanism causes attenuation of the exciting light beam and hence decreases the FL intensity of NS-CDs.^39^

To investigate the possibility of VLD sensing, the UV spectra of NS-CDs/Cu^+2^ were compared before and after the addition of VLD (Fig. S9†). The newly formed peak at 360 nm, corresponding to the formed complex, decreased in presence of VLD. This could be attributed to that VLD contains pyrrolidinecarbonitrile and aminoacetamide moieties which have metal complex formation ability.^40,41^ This serves in the competition between VLD and the synthesized NS-CDs for Cu^2+^ complexation, leading to a switchable off/on sensor for VLD detection.

### Determination of VLD and selectivity

**Fig. 4:**
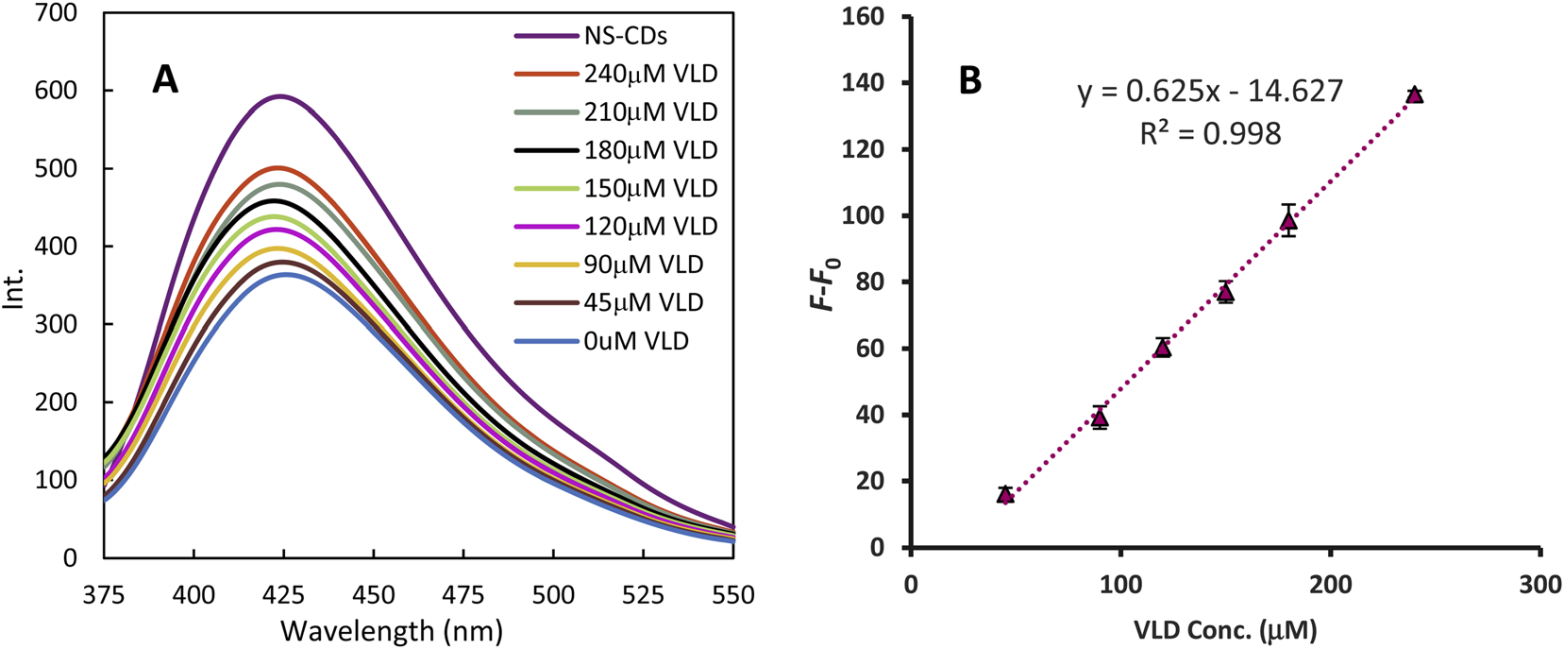
Fluorescence recovery of NS-CDs with increasing VLD concentrations (A), and linear relationship between fluorescence recovery and VLD concentration, where *F*_0_ and *F* are the fluorescence intensities of NS-CDs/Cu^2+^ and NS-CDs/Cu^2+^/VLD, respectively (B).

The NS-CDs FL was quenched by Cu^2+^ generating a sensor that was turned on sequentially with increasing VLD (Fig. 4). The FL recovery (*F*–*F*_0_) plot against VLD concentration demonstrated a linear calibration range of 45–240 μM (*r*^2^ = 0.998). The slope, intercept, and standard deviations were calculated using regression analysis and the detection limit (DL = 3.3 *σ*/*S*, where *σ* is the SD of the intercept of a calibration curve around the detection limit and *S* is the slope of the regression line) was estimated to be 13.411 μM (Table 1).

**Table 1:** Validation results for the synthesized sensor for VLD determination

Parameter	VLD
Linearity range (μM)	45–240 (13.65–72.82 μg mL^−1^)
Determination coefficient (*r*^2^)	0.998
Slope	0.625
Intercept	14.627
DL (μM)	13.411
QL (μM)	40.640
*S*_a_ (standard deviation of intercept)	2.168
*S*_b_ (standard deviation of slope)	0.014
*S*_y/x_ (standard deviation of residuals)	2.203

**Fig. 5:**
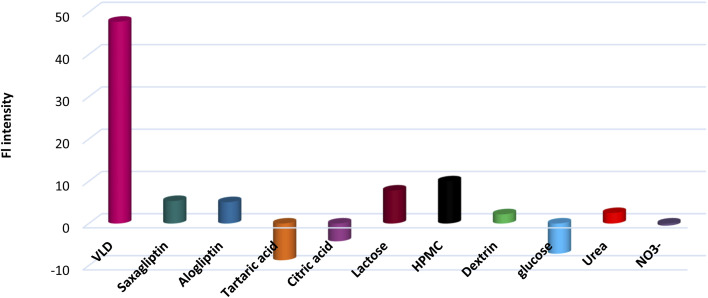
Response of NS-CDs/Cu^2+^ system towards VLD and possible interferants (saxagliptin, alogliptin, tartaric acid, CA, lactose, HPMC, dextrin, glucose, and nitrate); [alogliptin] = 25 μM, [urea] = 300 μM, [nitrate] = 25 μM and concentration of other analytes = 100 μM.

Interfering materials such as saxagliptin, alogliptin, tartaric acid, CA, lactose, HPMC, dextrin, urea, glucose, and nitrate were investigated to test the reliability of the developed sensing platform (Fig. 5). It was found that common species in pharmaceutical samples and urine in addition to some structurally related compounds exhibited negligible influence on VLD determination.

### Applications

#### Analysis of pharmaceutical preparation

The designed switchable sensor was used to quantify VLD in commercial tablets (50 mg VLD/tablet) relying on its excellent specificity, sensitivity, and quick response to VLD. Sample solutions were prepared and examined following procedures outlined in section “Tablet and urine sample analysis”. The results were presented in Table S1† and statistically compared to those obtained from the comparison method based on derivatization with 4-chloro-7-nitrobenzofurazan at 70 °C for 20 minutes.^11^ The statistical comparison revealed the sensor's reliability in determining VLD.

#### Analysis of urine samples

To further investigate the applicability and reliability of the proposed NS-CDs/Cu^2+^ sensor, the detection of VLD in human urine was performed. Urine samples were treated following the steps detailed in the experimental section “Tablet and urine sample analysis”. After that, samples were analyzed by the addition of a small aliquot of the treated urine samples into the NS-CDs/Cu^2+^ sensor, measuring FI, and using the calibration curve generated under the same treatment conditions. The calibration curve of VLD in urine was linear over the range (60–150 μM) with the following regression equation, *y* = 0.424*x* − 8.725 (*r*^2^ = 0.992). The results in Table 2 indicate good accuracy and precision of the developed sensor for the determination of VLD in urine matrix.

**Table 2:** Application of the proposed sensor for VLD analysis in human urine

Conc. added (μM)	Mean conc. found^a^ (μM) ± SD	Mean% recovery ± SD	% RSD
75.0	73.8 ± 1.43	98.4 ± 1.91	1.94
105.0	106.7 ± 1.19	101.6 ± 1.14	1.12
120.0	120.3 ± 1.24	100.2 ± 1.03	1.03

a *n* = 3.

## Conclusions

Herein, the development of a fluorescent switch off/on sensor for VLD determination was described based on ecofriendly, simple, quick, and affordable microwave NS-CDs preparation using CA and TSC. Cu^2+^ ions had the ability to significantly quench the FL of NS-CDs *via* a static quenching mechanism, where a non-fluorescent ground state complex was rapidly formed between Cu^2+^ and NS-CDs. In addition, the absorption spectrum of the formed complex overlapped with the excitation spectrum of NS-CDs causing attenuation of the exciting light beam of NS-CDs reducing its FL through the inner filter effect mechanism. Furthermore, the tendency of VLD to form a complex with Cu^2+^ could reduce the Cu^+2^ quenching effect on NS-CDs and hence turn on the sensor's FL. Consequently, this easy and label-free recognition system was used as the first CDs-based fluorescence determination of VLD letting it be used for routine analysis without the need for chemical derivatization. The sensor was successfully applied for vildagliptin determination in human urine samples with % recoveries from 98.4% to 100.2%.

## Compliance with ethical standards

The study involving human participant was performed in strict accordance with the institutional ethical standards of the Helsinki Declaration of 1964 and its later amendments. It was approved by the local research ethical committee of faculty of pharmacy, Tanta University, Egypt. Healthy volunteer was fully informed verbally about the objectives and nature of this study and a written informed consent was obtained from the volunteer involved in the study.

## Author contributions

Eman A. Elshenawy: conceptualization, methodology, investigation, validation, writing–original draft; Samah F. El-Malla: supervision, conceptualization, writing–review and editing; Sherin F. Hammad: supervision, conceptualization, resources, writing–review and editing; Fotouh R. Mansour: supervision, conceptualization, writing–review and editing.

## Conflicts of interest

There are no conflicts to declare.

## Supplementary Material

RA-012-D2RA04991K-s001
