# Supplementary material for: Innovative spectrofluorimetric determination of vildagliptin based on a “switch off/on” NS-doped carbon dot nanosensor
Source: RSC Adv. 2022 Sep 12;12(39):25815–21. doi: 10.1039/d2ra04991k (PMC9465401; doi:10.1039/d2ra04991k)
Supplement: RA-012-D2RA04991K-s001 [file RA-012-D2RA04991K-s001.pdf]

Electronic Supplementary Material (ESI) for RSC Advances.
This journal is © The Royal Society of Chemistry 2022

Supplementary Information

Innovative Spectrofluorimetric Determination of Vildagliptin Based on "Switch Off/On" NS-doped Carbon Dots Nanosensor

Eman A. Elshenawy [a*], Samah F. El-Malla [a], Sherin F. Hammad [a] & Fotouh R. Mansour [a]

[a] Department of Pharmaceutical Analytical Chemistry, Faculty of Pharmacy, Tanta University, Tanta, 31111, Egypt.

* Correspondence to eman.elshenawy@pharm.tanta.edu.eg

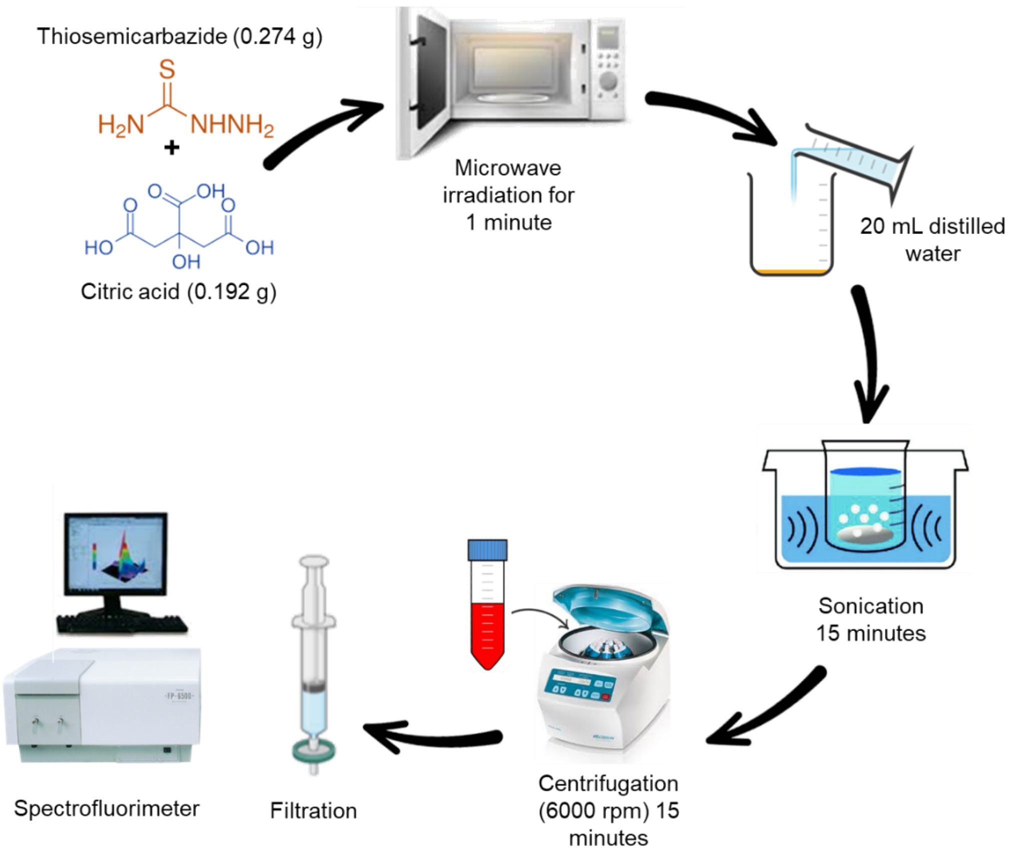

Scheme S1. Illustration of NS-CDs Synthesis

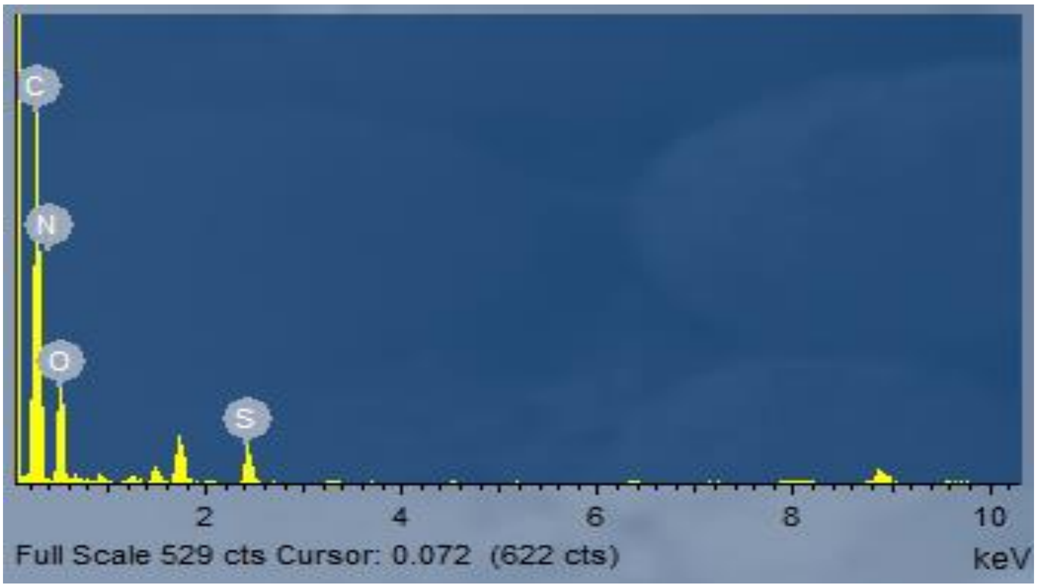

Fig. S1: TEM-EDX elemental analysis of the prepared NS-CDs.

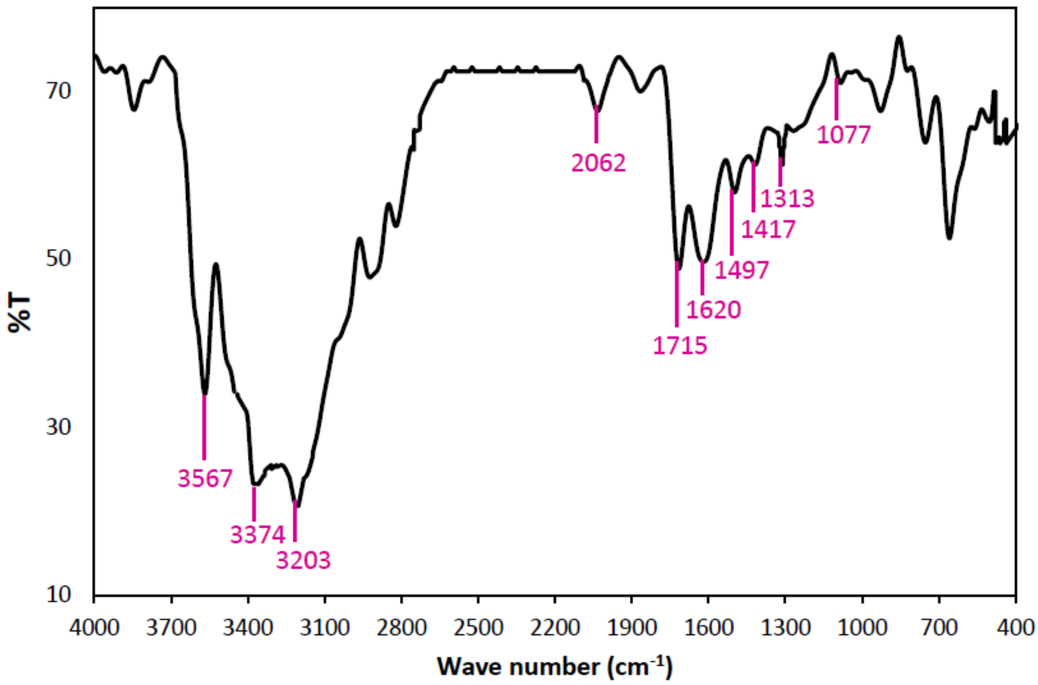

Fig. S2: IR spectrum showing the surface functional groups of the prepared NS-CDs.

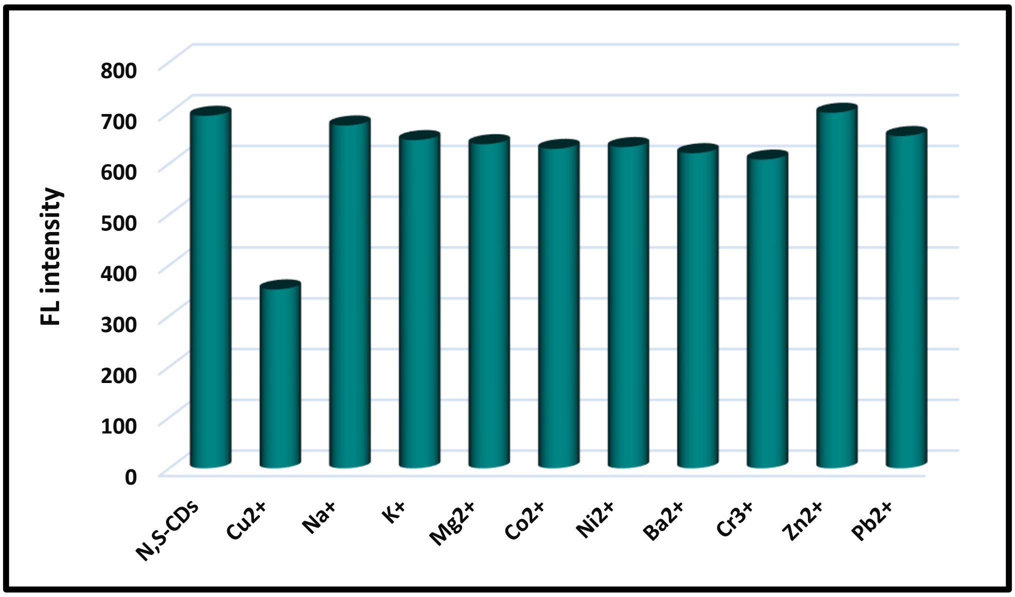

Fig. S3 Quenching performance of various cations (100 μM) on NS-CDs

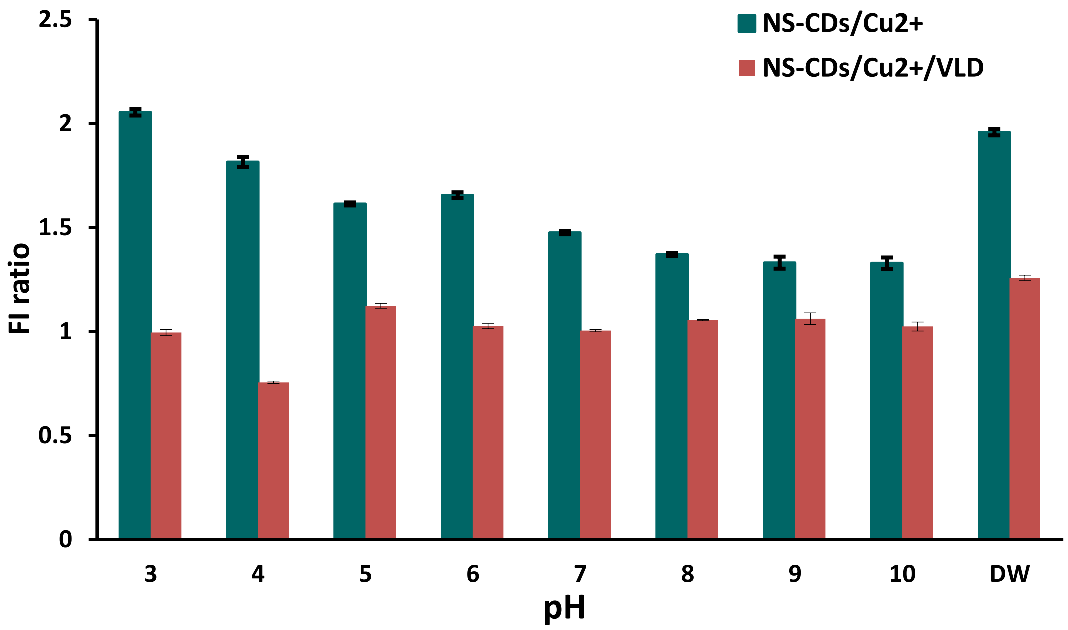

Fig. S4: The effect of pH on NS-CDs FL quenching by Cu^{2+}(where FI ratio is the ratio of FI of the NS-CDs and the FI of the NS-CDs/Cu^{2+} system) and FL recovery by VLD (where FI ratio is the ratio of FI of the NS-CDs/Cu^{2+}/VLD and the FI of the NS-CDs/Cu^{2+} system).

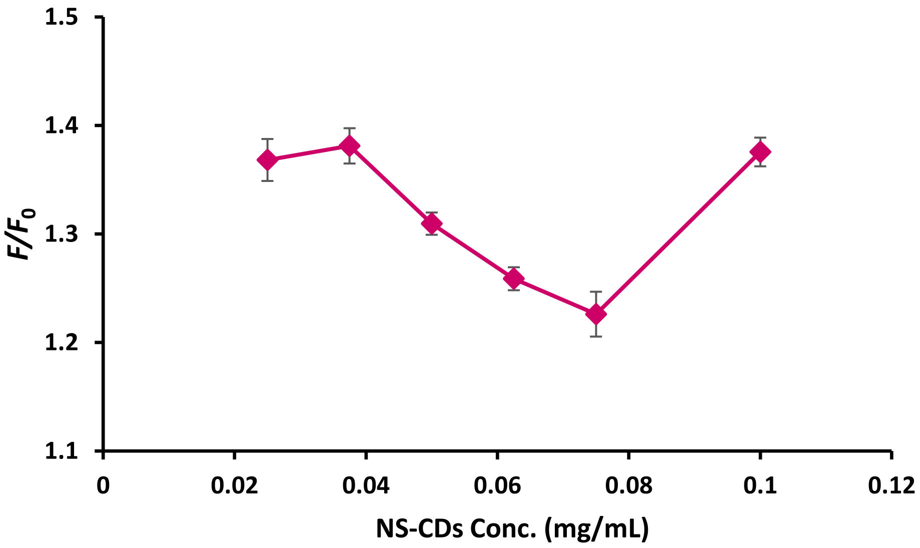

Fig. S5: The effect of NS-CDs concentration on VLD detection, where F_0 is FI of the NS-CDs/Cu^{2+} system and F is the FI of NS-CDs/Cu^{2+}/VLD system.

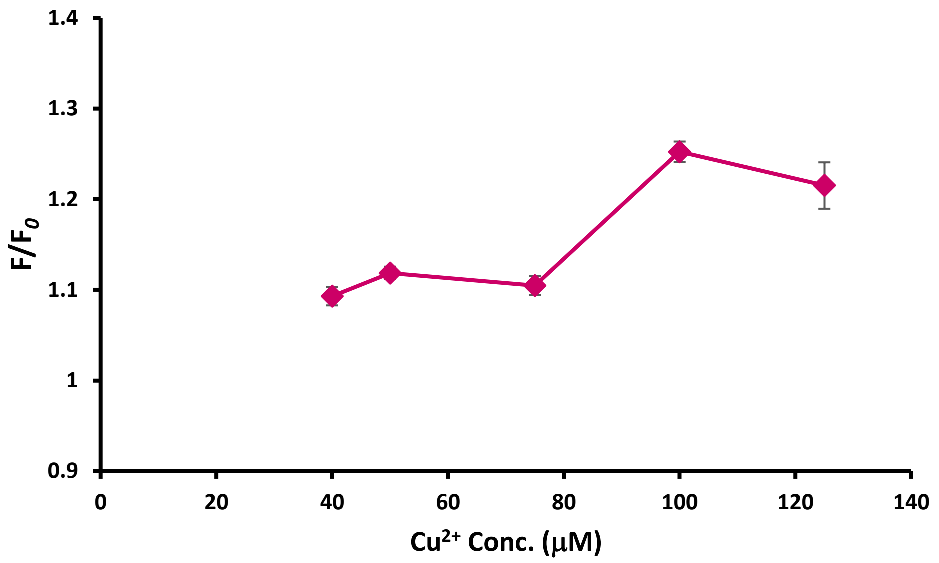

Fig. S6: The effect of Cu^{2+} concentration in NS-CDs/Cu^{2+} system on VLD detection, where F_0 and F are the FI of the NS-CDs/Cu^{2+} system and NS-CDs/Cu^{2+}/VLD system, respectively.

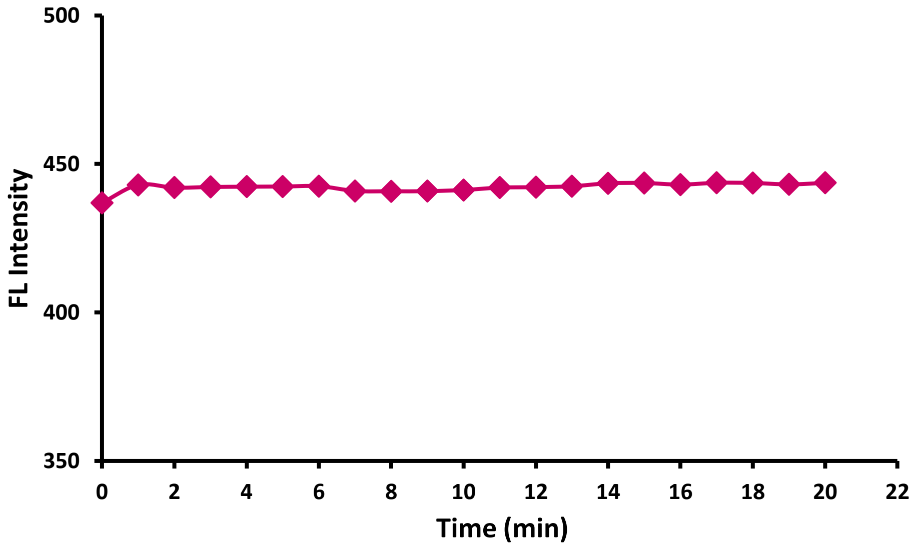

Fig. S7: The effect of reaction time on VLD determination by the developed NS-CDs sensor.

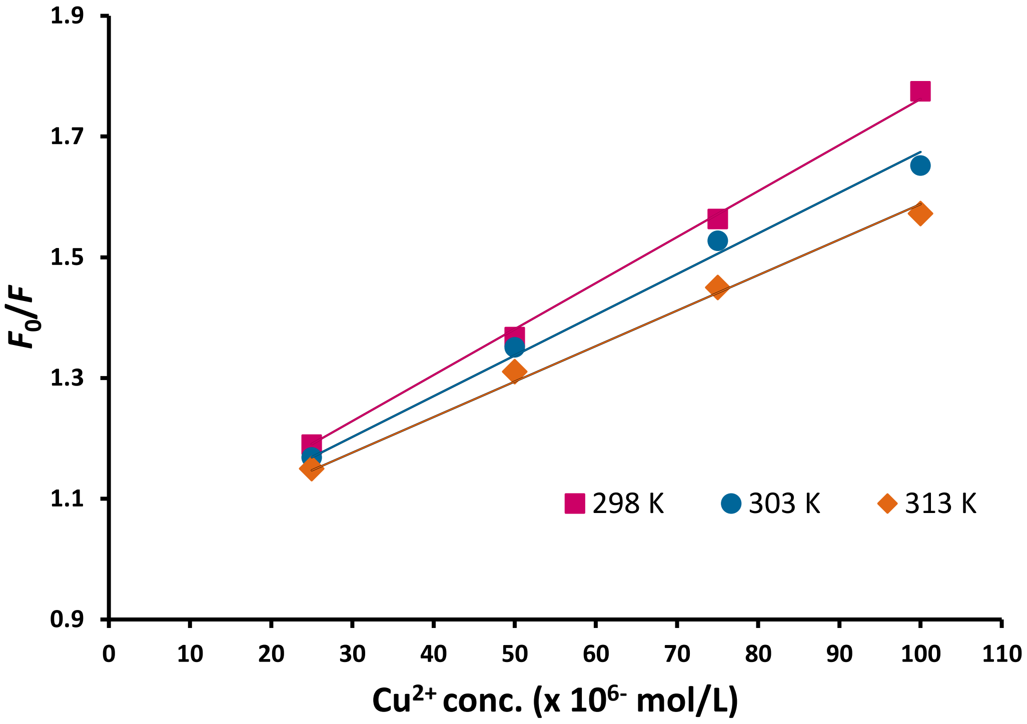

Fig. S8: Stern–Volmer plots for NS-CDs FL quenching by Cu^{2+}.

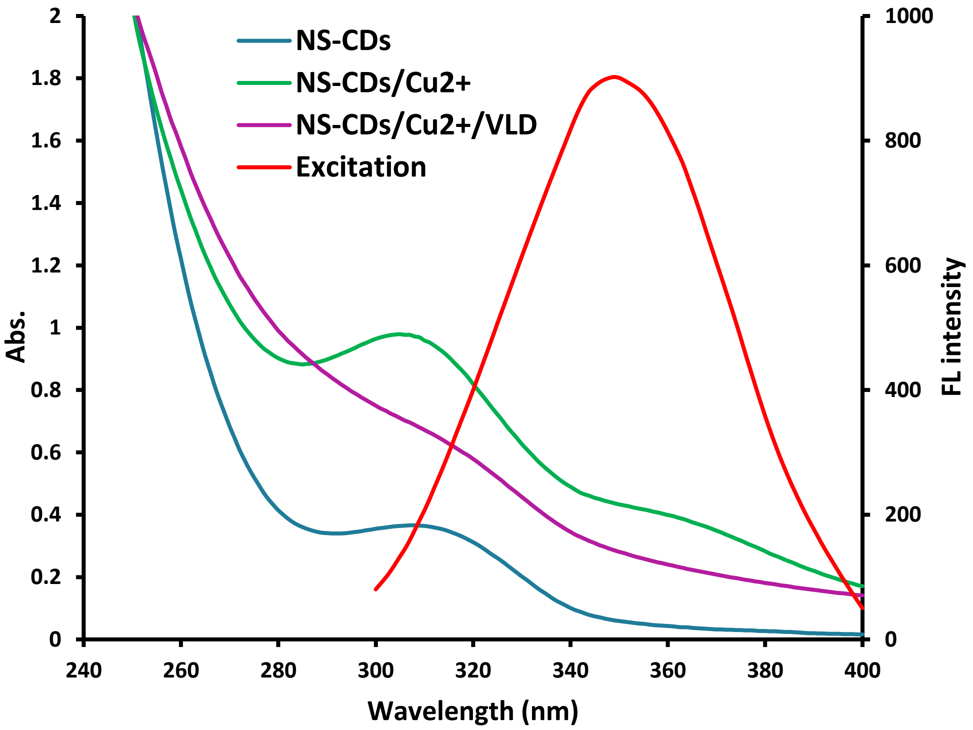

Fig. S9 Overlapping UV spectrum of NS-CDs/Cu2+ system with NS-CDs FL excitation and the effect of VLD addition on the system

Table S1 Application results for the synthesized sensor for analysis of VLD in marketed tablets.

**Vildagluse®** tablets	**Proposed method**		**Comparison method** [11]
	Conc. examined (μM)	Mean conc. found[a] (μM) ± SD	
	150.0	149.3 ± 0.25	
**Mean % recovery ± SD**		99.53 ± 0.30	99.7 ± 0.27
**%RSD**		0.30	0.27
***t*-test**		1.27 (2.228)	
***F*-test**		1.18 (5.050)	

[a] n= 6; tabulated *t* and *F* values at $\alpha = 0.05$.
